# Revised Chinese resident health literacy scale for the older adults in China: simplified version and initial validity testing

**DOI:** 10.3389/fpubh.2023.1147862

**Published:** 2023-05-17

**Authors:** Yilin Wang, Qiaoling Jia, Haiyan Wang, Kaiwen Zou, Lu Li, Bing Yu, Li Wang, Yanhong Wang

**Affiliations:** Department of Epidemiology and Biostatistics, Institute of Basic Medical Sciences, Academy of Medical Sciences and School of Basic Medicine Peking Union Medical College, Beijing, China

**Keywords:** health literacy, item response theory, differential item functioning, confirmatory factor analysis, older adults

## Abstract

**Objective:**

This study aimed to develop a short version of the Chinese Resident Health Literacy Scale focused on older adults in China, and further assess the reliability and validity of this short version.

**Methods:**

The data was from a cross-sectional community-based older adults health survey conducted in 2020. The total of 5,829 older adults were randomly divided into two parts using for the simplification and assessment of the scale, respectively. Item Response Theory (IRT) and Differential Item Functioning (DIF) were used for item analysis and scale simplification. Cronbach’s alpha and McDonald’s omega were used to assess the reliability and three factors Confirmatory Factor Analysis (CFA) was used to assess the validity, which were compared to the original version. Moreover, Multi-group Confirmatory Factor Analysis (MCFA) was used to test the model invariance of the short version across groups of gender, age groups, level of education, and cognitive status.

**Results:**

The simplified version consisted of 27 items taken from 50 original items, of them 11 items from the dimension of knowledge and attitudes, 9 items from the dimension of behavior and lifestyle, and 7 items from the dimension of health-related skills. The overall Cronbach’s alpha and McDonald’s omega were both 0.87 (95%CI: 0.86–0.88). The goodness-of-fits of CFA in simplified version were still acceptable in CFI, TLI, GFI, and RMSEA, even improved in CFI, TLI, and GFI compared to those of original version. Also, the model was stable and invariant in MCFA across gender, cognitive status, and educational level groups.

**Conclusion:**

In this study, we formed a simplified instrument for measuring health literacy focused on older adults in China. This short version might be more suitable for the priority recommendation in extended tracking of the dynamic changes on the levels of health literacy in the whole life cycle in public health settings. Further research might be to identify the cut-off values to distinguish the older adults with different levels of health literacy.

## Introduction

Among community-dwelling older adults, inadequate health literacy was independently linked to poorer physical and mental health (1). The previous studies found that lower health literacy was related to less understanding health information, a lack of basic knowledge of diseases, and/or poorer medication adherence (2), which could further increase the hospitalization and mortality rates (3, 4). Especially for those suffering from chronic diseases, over 40% of them took a grave risk to misunderstand, forget, or ignore healthcare advice (5). Therefore, promoting health literacy might be the most effective and affordable strategy for dealing with Non-communicable Chronic Disease (NCD) challenges (6, 7). In order to develop interventions to improve health literacy, it needed to give priority to measure the level of health literacy, especially among older adults, which was fundamental for the evaluation and surveillance.

Until now, over 150 instruments of health literacy have been developed, ranging from traditional tools focused on individual skill and health education to updated instruments from multidimensional perspectives (8–11). In China, health literacy also attracted more and more attention and some instruments were developed and applied in clinical or public health settings (12–17). Of these instruments, the Chinese Resident Health Literacy Scale (CRHLS) was developed on the manual of “Basic Knowledge and Skills of People’s Health Literacy” published by the National Health and Family Planning Commission and firstly released in 2008 (16). It was widely used in the National Health Literacy Surveillance among Chinese residents aged 15–69 years old (17, 18). According to data from National Health Literacy Surveillance, the health literacy levels (CRHLS scores of 80% or above) increased to 23.15% in 2020 and social development index, age, and education level were highly associated with health literacy (19). However, rare data of the health literacy for the older adults were released and in previous published study only 5.31–7.74% older adults aged 60–69 were found to reach this level (20).

CRHLS might be unsuitable for older adults in China. Firstly, the time consuming might be a huge challenge for older adults. Most of the young and middle-aged adults might spend about 30 min to complete measurement (16), while older adults might need to spend more time to do it, even longer for those with limited literacy (21). Secondly, some of items in CRHLS might be floor or ceiling effects for older adults. For example, the items of “reading and understanding OTC drug facts label” or “description of the liver function” might be beyond the scope of reading and understanding ability especially for those with lower level of education, or some items (e.g., item of “national unified toll-free hotline number”) deviated from the areas of concern for some older adults, which might led to the lower awareness rates. Lastly, CRHLS score 80% or more as the cutoff value with health literacy might be too strict for older adults and lack of evidence (22). Developing a brief version adapted from the original CRHLS for older adults might have several advantages, including brevity, availability of normative data, ease of administration, and lower cost. It would be more suitable for tracking the dynamic changes in the whole life cycle and evaluating the relationship between health literacy and health outcomes or health behaviors in the Chinese society context. Therefore, it was necessary to investigate how to simplify original scale reasonably and maintain the original reliability and validity as much as possible.

Item response theory (IRT) was a complex approach that attempted to explain the relationship between latent traits (unobservable characteristic or attribute) and their manifestations (i.e., observed outcomes, responses or performance) (23–31). Recently, more and more researchers used IRT models to analyze the latent properties of the scales and furtherly revise or simplify the scales (32). The advantage of using IRT was that each item in the scale was paid attention during the simplification of scale. It was assumed that the latent construct (e.g., knowledge, attitudes, *et al*) and items measured were organized in an unobservable continuum (24), which established a link between the properties of items on an instrument, individuals responding to these items and the underlying trait being measured (23).

Because that the level of education and cognitive status were the important factors related to the health literacy in previous studies (33, 34), and the health literacy level of older adults associated positively with the education level or cognitive function. The equivalence in the measurement of health literacy should be considered across groups of cognitive ability or education. The differential item functioning (DIF) to assess the equivalence of items in the scales was more and more applicated in some studies of educational or health assessment (35). The measurement invariance (MI) was defined as statistical property of a measurement that the same underlying construct was measured across groups or across time (36). MI should be considered in the simplification of scale and lack of MI (meaning DIF) indicated that the populations with the same latent ability do not have equal probability of getting an item correct, regardless of group membership (37). DIF also examined the relationship between the item response and another group variable, after controlling for the underly construct (38). When groups had different probabilities of response to a given item in DIF analysis, that indicated DIF occurred and this item was labeled as DIF-item (37).

Therefore, the purpose of this study was to simplified CRHLS based on IRT and DIF methods to form a more suitable instrument of health literacy for the Chinese older adults, and further assess the reliability and validity of simplified version.

## Methods

### Participants

The data was from a cross-sectional health survey carried out in Beijing among the community-based older adults in 2020, in which 5,829 participants were used for analysis in this study (39).

The simple random sampling method was used to select the 50% of total older adults as the sample for simplification (marked as Sample A) and the other 50% as the sample for validation (marked as Sample B). There were no differences in demographic and original version health literacy scores found between the two samples.

### Measures

The survey collected information on sociodemographic characteristics (including age, gender, ethnicity, education level, marital status, and so on), health literacy, and cognition impairment.

Health literacy was measured by the CRHLS for the 2018 edition, which consisted of 50 items to assesses health knowledge, attitudes, behaviors and skills that are necessary to address real-world health problems and three dimensions: (1) knowledge and attitudes: basic knowledge and concepts related to health (22 items); (2) behavior and lifestyle: health-related behaviors and lifestyle (16 items); and (3) health-related skills: basic health-related skills (12 items). There are four types of questions in the scale: true-or-false; single-answer (only one correct answer in multiple-choice questions); multiple-answer (more than one correct answer in multiple-choice questions); and situational questions. With multiple-answer questions, a correct response had to contain all the correct answers and no wrong ones. Situational questions were given following a paragraph of instruction or medical information. Of these items, according to the scoring rules of this scale 2018 edition, total 50 items were included in the scores of health literacy with correct response allocated 1 point (except for the multiple-answer items in which correct response was allocated 2 points). The total score ranged from 0 to 66 points. Cronbach’s α coefficient and the McDonald’s omega of the Chinese Resident Health Literacy Scale were both 0.90 in our study.

Older adults might experience subtle cognitive changes associated with aging. The Ascertain Dementia 8 (AD8) questionnaire was used for screening cognitive impairment in this study (40). AD8 was a brief informant-based measure with only eight questions and performed well in distinguishing cognitive impairment from normal cognition (41). The person with an AD8 score ≥ 2 was suspected of having a cognitive impairment and needed further testing to be diagnosed. Cronbach’s α coefficient of AD8 was 0.87 in our study.

### Data analysis

#### The first phase of simplification in sample A

There were two stages included in this study. The first phase was used for simplification of the CRHLS in Sample A. The two-parameter logistic model (2PLM), one of the IRT models, was used to predict the probability of a successful answer for each item in each dimension (40). Difficulty and discrimination were two important parameters in 2PLM. The formula was as follows (42):


$$P_{\left(x_{j}=1|\theta \right)=\frac{1}{1+\exp\left[-a_{j}\left(\theta -b_{j}\right)\right]}}$$

Where $x_{j}$ was the response to item j in the scale; $a_{j}$ was the discrimination parameter; $b_{j}$ was the difficulty parameter, which indicated the point where an individual would have a 50% chance of endorsing item j ; and θ is the ability value being measured. The basic assumptions of IRT included unidimensionality, local independence, and monotonicity (25–29), which were assessed, respectively, by the eigenvalue examination in factor analysis (30), Yen’s Q3 statistic (29), splines modeling via flexible IRT models and Pearson *χ*^2^ statistics (31) in this study.

The information about individuals was based on their responses to items and the properties of all the items (43). The maximum likelihood method was used for parameter estimations. According to the parameters of discrimination and difficulty, the information function for each item was calculated (44). Item information functions for the 2PLM would have their maximum value at the value of the threshold (difficulty) (16). And slopes (discrimination) control how peaked the item information function was, and the higher the slope value was, the more information that item provided, the smaller the measurement error (45). In our study, the item response probability was chosen as 2/3 to calculated θ value in order to estimate the maximum amount of information for each item labeled as I(θ), which was one of the most popular standard setting methods of response probability in the Bookmark method (46–48).

Furthermore, in order to present how each dimension of scale was functioning as a whole, we also combine the item-level information functions to create a test information function, shown as test information curves (TIC). The standard errors were the square root of the inverse of information at a given level of ability. The item, with a discrimination parameter of 0.5 to 2.0 and a difficulty parameter of −3.0 to 3.0, was considered to provide the most information (16), which was also used in this study. Furthermore, I(θ) was also the criteria of item selection in this study, that was: removed items (I(θ) ≤ 0.10), modified items (0.10 < I(θ) ≤ 0.20), and kept items ((I(θ)>0.20) (49).

Moreover, Differential Item Functioning (DIF) was used to test the equivalence of items. We applied logistic regression for identification of DIF using the ability (θ) derived from IRT analysis as the matching variable. This method has been used in previous published studies (50–52). The group variables were cognitive status (Normal or Impairment) or the levels of education (Illiterate or Primary school, Junior high school, and Senior high school or above). If the determination coefficient (*value of p*) of DIF for a given item was significant (item with DIF), the item was labeled as DIF-item, which was considered to be removed from the scales (53).

In brief, if any of the following criteria was met in this phase, the item would be removed from original CRHLS: (a) discrimination(α) <0.5 or > 2.0 (16); or (b) difficulty(b) < −3.0 or > 3.0 (16); or (c) I(θ) ≤0.20 (49); or (d) items exhibiting DIF on cognitive status or education level (54).

#### The second phase of validation in sample B

The second phase was validation of the simplified version as above in Sample B. Compared to the original version, the reliability, the construct validity, as well as Goodness-of-fit was estimated in the simplified version scale. Both Cronbach’s alpha and McDonald’s omega, as well as their 95%CI, were used to assess the reliability of the simplified version scale. The three factors Confirmatory Factor Analysis (CFA) was used to assess the convergent validity and discriminant validity. The goodness-of-fit was examined via structural equation modeling using the maximum likelihood method (55). The indicators of comparative fit index (CFI), Tucker-Lewis index (TLI), goodness-of-fit index (GFI), and root of the mean square residual (RMSEA) were calculated and compared between models. It was considered to be acceptable for the goodness-of-fit when CFI > 0.90, TLI > 0.90, GFI > 0.90, and RMSEA<0.08 (56).

In addition, Multi-group CFA (MCFA) was used to test the invariance of the simplified version across groups of gender, age, level of education, and cognitive status. The four models were used in MCFA analysis, that was: (1) configural invariance: the basic factor structure was the same across groups; (2) metric invariance: constraining factor loadings to be equal between groups based on the basic factor structure; (3) strong invariance: constraining the intercepts of the items to be the same between groups based on metric invariance; (4) strict invariance: further constraining residuals to be equal between groups (57). Invariance across subgroups is depicted by significant χ^2^, chi-square should not significantly differ between models, along with ΔCFI<0.02, ΔTLI<0.02, and ΔRMSEA<0.015 (58).

IRT analyses were conducted using the R mirt package (59). The calculation of reliability was conducted using the R ltm and coefficientalpha packages (60). CFA and MCFA were performed in AMOS 17.0. Other analyses, including descriptive analysis, t-test, and chi-square test, were conducted using SAS 9.4. The significance level was 0.05 for all statistical tests.

## Results

A total of 5,829 older adults were included in this study, 50% of male, mean age of 70.29 ± 7.37 years old, about 23% from rural regions, and 28% of education level of primary school or below. In total, 1888 participants (32.39%) might be cognitive impairment screened by AD8 (scores of AD8 ≥ 2 points). There was no significant difference in the characteristics between Sample A and Sample B (*p* > 0.05; Table 1).

**Table 1:** Characteristics of study participants.

Demographic variables	Total (*N* = 5,829)	Simplification (Sample A, *N* = 2,915)	Validation (Sample B, *N* = 2,914)	*p*-value
*Gender*				
Male	2,891 (49.6%)	1,448 (49.67%)	1,443 (49.52%)	0.91
Female	2,938 (50.4%)	1,467 (50.33%)	1,471 (50.48%)	
Subgroups of age				
60–69	2,925 (50.18%)	1,473 (50.53%)	1,452 (49.83%)	
70–79	2,166 (37.16%)	1,077 (36.95%)	1,089(37.37%)	0.65
≥80 or over	738 (12.66%)	365 (12.52%)	373(12.80%)	
Ethnicity				
Han	5,614 (96.3%)	2,811 (96.43%)	2,803(96.19%)	0.63
Other	215 (3.7%)	104 (3.57%)	111(3.81%)	
Type of residence				
Urban	4,505 (77.29%)	2,252 (77.26%)	2,253(77.32%)	0.96
Rural	1,324 (22.71%)	663 (22.74%)	661(22.68%)	
Education				
Illiterate or Primary school	1,625 (27.88%)	794 (27.24%)	831(28.52%)	
Junior high school	2,129 (36.52%)	1,056 (36.23%)	1,073(36.82%)	0.65
Senior high school or above	2075 (35.60%)	1,065 (36.54%)	1,010(34.66%)	
Marital status				
Married	4,856 (83.30%)	2,441 (83.74%)	2,415(82.88%)	0.38
Unmarried, Divorced, or Widow	973 (16.69%)	474 (16.26%)	499(17.12%)	
Smoking condition				
Never Smoking	3,855 (66.13%)	1,951 (66.93%)	1904 (65.34%)	
Smoking	1,155 (19.81%)	562 (19.28%)	593(20.35%)	0.25
Smoked, but quit now	819 (14.05%)	402 (13.79%)	417(14.31%)	
Medical insurance				
Yes	5,721 (98.15%)	2,852 (97.84%)	2,869(98.46%)	0.09
No	108 (18.34%)	63 (2.16%)	45(1.54%)	
Cognitive ability				
Cognitive normal	3,941 (67.60%)	1978 (67.86%)	1963(67.36%)	0.69
Cognitive impairment	1,888 (32.40%)	937 (32.14%)	951(32.64%)	
Total scores of health literacy (Mean ± SD)	39.20 ± 12.80	39.31 ± 12.69	39.08 ± 12.91	0.49
Scores of three dimensions (Mean ± SD)				
Knowledge and attitudes	17.24 ± 5.57	17.27 ± 5.53	17.20 ± 5.61	0.63
Behavior and lifestyle	12.79 ± 4.90	12.83 ± 4.89	12.74 ± 4.91	0.51
Health-related skills	9.17 ± 3.61	9.22 ± 3.57	9.13 ± 3.64	0.34
Health literacy*				
Less than 80% of total scores	4,921 (84.4%)	2,460(84.4%)	2,461(84.50%)	0.97
80% of total scores or above	908 (15.6%)	455(15.6%)	453(15.50%)	

*The scores of 80% or more was the cutoff value with health literacy in CRHLS.

### The simplification of Chinese resident health literacy scale

As shown in Table 2, the final 27 items kept in the simplified version of health literacy for older adults based on IRT and DIF consisted of 11 items in the knowledge and attitudes dimension, 9 items in the behavior and lifestyle dimension, and 7 items in the health-related skills dimension. A total of 23 items were removed, of which 21 items based IRT with low information or discrimination, and/or 4 DIF-items on cognitive status or education level (Table 2).

**Table 2:** Evaluation of items based on IRT and results of DIF analysis of the original version of the Chinese Resident Health Literacy Scale in Sample A.

Item abbreviation		Correct (%)	Correlation to dimension score	IRT parameters			DIF						Kept
							Cognitive Status			Education Level			
				Difficulty	Discrimination	Information level	Statistic	*p*	*R*^2^	Statistic	*p*	*R*^2^	
Dimension1: Knowledge and attitudes													
A01	Prevention of the flu	79.35	0.43	−1.44	1.13	0.32	0.52	0.74	0.0001	0.65	0.52	0.0002	Yes
A02	Health care products	79.20	0.26	0.58	1.42	0.08	<0.001	0.99	<0.0001	5.20	0.07	0.0017	No
A03	Infusion	79.86	0.42	−1.43	1.08	0.29	0.22	0.76	0.0001	0.64	0.52	0.0002	Yes
A05	Health products	79.11	0.31	−2.26	0.63	0.10	<0.001	0.99	<0.0001	5.20	0.07	0.0017	No
A10	Body temperature	78.03	0.23	−3.57	0.37	0.03	7.90	0.04	0.0026	7.16	0.78	0.0002	No
B01	The definition of health	71.90	0.52	−0.92	1.43	0.51	0.20	0.76	<0.0001	4.63	0.08	0.0011	Yes
B03	Hepatitis B	56.30	0.38	−0.96	0.75	0.14	0.99	0.63	0.0003	3.87	0.05	0.0024	No
B04	Blood pressure measurement	48.75	0.38	−0.40	0.69	0.12	0.79	0.63	0.0002	0.03	0.78	0.0003	No
B06	Early symptoms of cancer	51.32	0.41	0.11	0.82	0.17	2.27	0.32	0.0006	5.71	0.90	<0.0001	No
B07	Management of gas poisoning	77.12	0.40	−1.55	0.93	0.21	4.91	0.08	0.0014	2.51	0.19	0.0007	Yes
B08	Tuberculosis treatment	61.68	0.57	−0.05	1.55	0.59	8.59	0.04	0.0019	4.38	0.10	0.0011	No
B09	Toxic and hazardous work	70.60	0.47	−0.96	1.16	0.34	6.48	0.05	0.0017	4.72	0.08	0.0013	Yes
B10	Harm of iodine deficiency	64.01	0.36	−0.79	0.63	0.10	0.09	0.80	<0.0001	0.27	0.87	<0.0001	No
B13	Vaccines for children	63.29	0.45	−0.69	0.93	0.19	0.37	0.76	0.0001	0.44	0.07	0.0016	No
B17	Meaning of warning diagram	69.47	0.51	−0.79	1.35	0.45	3.98	0.13	0.0010	5.25	0.07	0.0013	Yes
C02	Medical visits	70.31	0.45	−0.98	1.08	0.29	0.27	0.76	0.0001	6.20	0.07	0.0017	Yes
C03	Liver	14.30	0.45	−1.49	1.35	0.46	14.06	0.00	0.0039	7.69	0.08	0.0010	No
C06	Packaged food	37.29	0.28	2.75	0.73	0.13	0.89	0.63	0.0003	0.93	0.66	0.0001	No
C07	Treatment of sick and dead livestock	68.27	0.50	−0.70	1.22	0.37	1.04	0.64	0.0003	0.04	0.87	<0.0001	Yes
C15	Pesticide storage	58.44	0.49	−0.38	1.13	0.32	5.13	0.08	0.0013	8.44	0.06	0.0021	Yes
D03	Control weight	61.64	0.48	−0.55	1.07	0.28	0.14	0.78	<0.0001	0.77	0.52	0.0002	Yes
D04	Obesity-related disease	67.79	0.46	−0.86	1.07	0.27	6.27	0.05	0.0017	2.22	0.21	0.0006	Yes
Dimension2: Behavior and lifestyles													
A04	Fruits and vegetables	80.75	0.37	−2.17	0.70	0.12	0.35	0.74	0.0001	0.75	0.78	0.0001	No
A06	Depression	86.24	0.36	−2.33	0.88	0.19	4.80	0.23	0.0015	1.07	0.05	0.0022	No
A09	Chronic disease treatment	62.95	0.33	−1.37	0.41	0.04	1.42	0.64	0.0005	0.04	0.78	0.0001	No
B05	Dangers of smoking	52.66	0.50	−0.05	0.99	0.24	2.38	0.49	0.0006	1.46	0.73	0.0004	Yes
B11	Hydration	62.57	0.46	−0.63	0.84	0.17	0.08	0.89	<0.0001	0.49	0.78	0.0002	No
B12	National basic public health service	40.07	0.49	0.51	1.05	0.27	0.28	0.74	0.0001	2.93	0.67	0.0008	Yes
B14	Fever	77.43	0.4	−1.80	0.80	0.15	1.11	0.64	0.0003	0.23	0.90	<0.0001	No
B15	Adverse reactions	79.52	0.33	−2.30	0.62	0.09	0.03	0.89	<0.0001	0.49	0.26	0.0008	No
B19	Medical visits	71.18	0.62	−0.91	1.29	0.41	0.84	0.64	0.0002	0.15	0.80	<0.0001	Yes
B21	Opening windows for ventilation during flu season	70.05	0.45	−0.98	0.99	0.24	0.88	0.64	0.0002	0.53	0.78	0.0001	Yes
C01	Promoting mental health	52.25	0.58	0.03	1.92	0.90	1.34	0.64	<0.0001	1.86	0.69	<0.0001	Yes
C04	Fever and rash in children	56.09	0.6	−0.17	2.00	0.97	0.47	0.74	<0.0001	2.34	0.67	<0.0001	Yes
C09	Benefits of eating soy products	31.60	0.43	0.95	1.05	0.27	2.63	0.49	0.0007	0.24	0.78	0.0001	Yes
C10	Health benefits of exercise	45.11	0.54	0.23	1.62	0.63	0.02	0.89	<0.0001	0.01	0.96	<0.0001	Yes
C12	Coughing and sneezing	46.90	0.38	0.29	0.53	0.07	5.75	0.23	0.0018	5.89	0.26	0.0005	No
C13	Medical visits	67.75	0.52	−0.72	1.21	0.36	0.41	0.74	0.0001	0.25	0.78	0.0001	Yes
Dimension3: Health-related skills													
B16	Treatment of virulent infectious diseases	83.81	0.44	−1.63	1.24	0.36	0.03	0.93	<0.0001	0.14	0.26	0.0007	Yes
B18	Toll-free health hotline number	34.90	0.41	1.00	−0.75	0.24	2.63	0.27	0.0007	0.07	0.01	0.0033	No
B20	OTC	28.37	0.42	1.38	0.79	0.15	2.54	0.27	0.0007	0.24	0.42	0.0002	No
B22	Glass thermometer	62.74	0.44	−0.79	0.7	0.12	0.20	0.85	0.0001	0.28	0.49	0.0003	No
B25	Mild burns	73.21	0.45	−1.31	0.92	0.19	2.10	0.29	0.0006	0.05	0.10	0.0010	No
B26	Fire handling	46.14	0.4	0.41	0.55	0.07	0.01	0.93	<0.0001	0.37	0.24	0.0018	No
C08	Cardiac arrest	59.07	0.52	−0.31	1.09	0.28	4.24	0.16	0.0011	0.10	0.36	0.0004	Yes
C11	Hypoglycemic products	70.94	0.47	−1.03	0.99	0.23	7.51	0.07	0.0020	0.41	0.26	0.0006	Yes
C14	Benefits of breastfeeding for babies	44.94	0.52	0.24	1.13	0.30	0.34	0.84	0.0001	2.64	0.26	0.0006	Yes
C16	Lightning weather outdoors	82.40	0.42	−1.80	0.99	0.23	0.61	0.75	0.0002	0.04	0.42	0.0002	Yes
D01	Calculation of BMI	35.13	0.54	0.61	1.62	0.63	6.43	0.07	0.0013	0.05	0.14	0.0011	Yes
D02	Classification of BMI	42.50	0.56	0.28	1.69	0.69	0.14	0.85	<0.0001	1.22	0.26	<0.0001	Yes

*R*^2^: DIF magnitude, applying the cut-offs of: <0.13 = negligible; 0.13–0.26 = moderate; >0.26 = not negligible (53).

### The reliability and validation of simplified version

Due to the 23 items removed, the overall Cronbach’s alpha coefficient for simplified version was 0.87 (95%CI: 0.86–0.88), slightly lower than that of original version (0.90, 95%CI: 0.89–0.90), but still considered better reliability (Table 3). The sufficient reliabilities were also found in the dimension of knowledge and attitudes (0.72, 95%CI: 0.70–0.73) and behavior and lifestyle (0.73, 95%CI: 0.72–0.75), while undesirable reliability in dimension of health-related skills (0.65, 95%CI: 0.63–0.67). There was a similar McDonald’s omega between simplified version and original version (Table 3). Also, the characteristics of items, the correlation to dimension scores, and factor loading in the simplified version in Sample B were detailed in Supplementary Table 1.

**Table 3:** Reliability of the original version and the simplified version of the Chinese Resident Health Literacy Scale in Sample B.

Dimension	Cronbach’s alpha	95%CI	McDonald’s omega	95%CI
Original version				
Overall	0.90	0.89–0.90	0.90	0.89–0.90
Three dimensions				
*Knowledge and attitudes*	0.78	0.77–0.80	0.79	0.78–0.80
*Behavior and Lifestyle*	0.74	0.73–0.75	0.74	0.73–0.76
*Health-related Skills*	0.70	0.68–0.71	0.70	0.68–0.72
Simplified version				
Overall	0.87	0.86–0.88	0.87	0.86–0.88
Three dimensions				
*Knowledge and attitudes*	0.72	0.70–0.73	0.72	0.70–0.73
*Behavior and Lifestyle*	0.73	0.72–0.75	0.74	0.72–0.75
*Health-related Skills*	0.65	0.63–0.67	0.65	0.62–0.67

**Figure 1:**
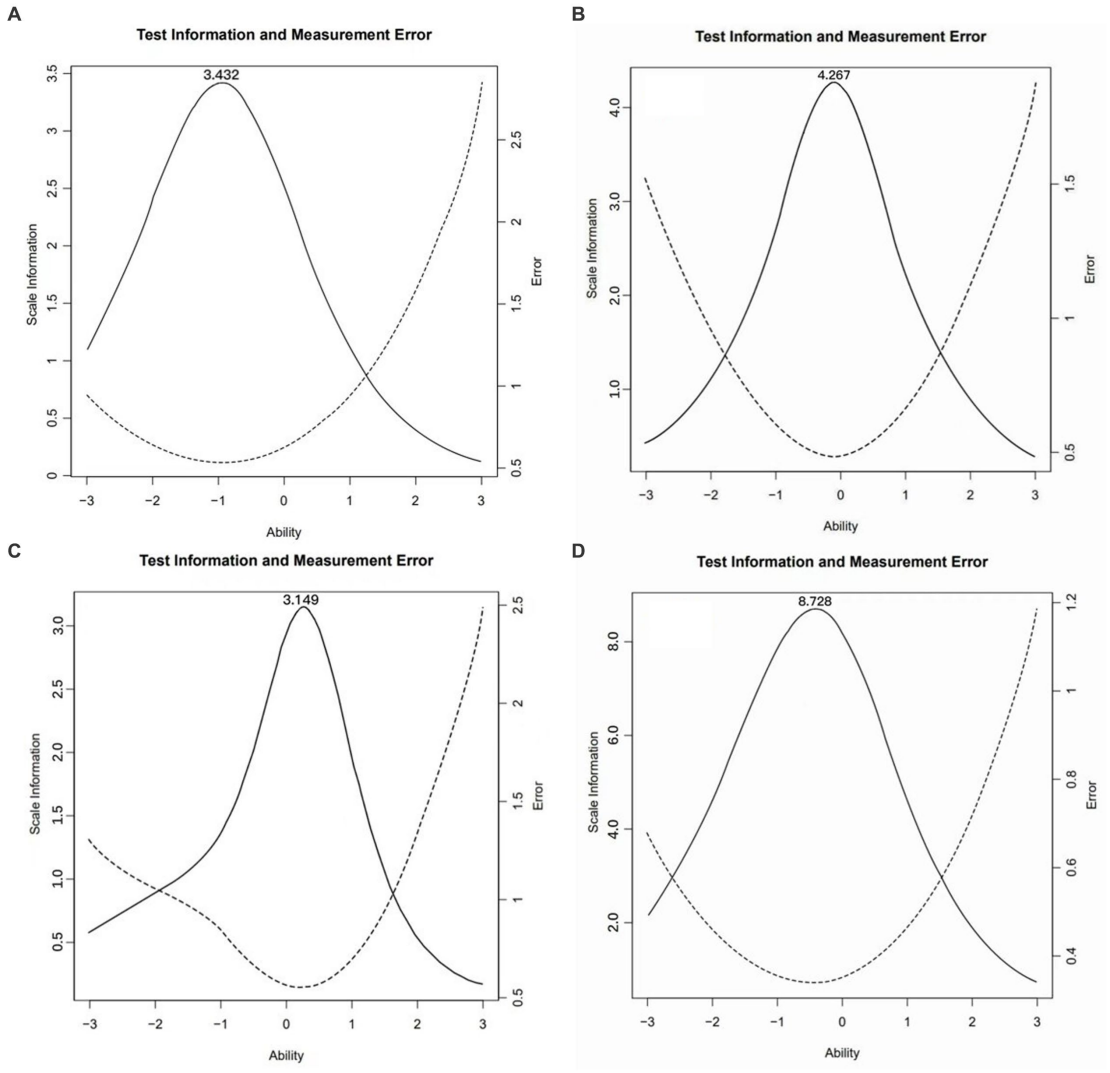
The test information and measurement error curves in simplified version. **(A)** Dimension of knowledge and attitudes; **(B)** Dimension of behavior and lifestyle; **(C)** Dimension of health-related skills; and **(D)** the total of simplified version.

The results in Table 4 indicated the goodness-of-fits of CFA. The indicators of three-factor structures in simplified version were still acceptable in CFI, TLI, GFI, and RMSEA, even improved in CFI, TLI, and GFI compared to those of original version. The test information and measurement error curves were shown in Figure 1.

**Table 4:** Goodness-of-fit of the CFA models of the original version and the simplified version of the Chinese resident health literacy scale in Sample B.

	*χ*^2^	df	*p* (*χ^2^*)	CFI	TLI	GFI	RMSEA	RMSEA 95%CI
Original version	9420.34	1,171	<0.001	0.693	0.679	0.852	0.049	0.048–0.050
Original version (modified)^*^	8625.16	1,166	<0.001	0.742	0.728	0.866	0.041	0.040–0.043
Simplified version (modified)^**^	3099.31	319	<0.001	0.825	0.808	0.910	0.055	0.054–0.056

CFI, Comparative fit index; TLI, Tucker-Lewis index; GFI, Goodness of Fit Index; RMSEA, root mean square error of approximation.

*modified original version with five specified error covariances: A01 with A03; A09 with A10; B18 with B20; C06 with C10; D01 with D02.

**modified simplified version with two specified error covariances: A01 with A03; D01 with D02.

### Testing for measurement invariance of simplified version

After then, Sample B was stratified by gender (male or female), age (60–70 years or ≥ 70 years or over), cognitive status (normal or impairment), or education level (illiterate or primary school, junior school, or high school or above), then fitted into the model of the simplified version separately.

As shown in Table 5, the model of the simplified version is invariant and stable across groups of gender, age, cognitive status, and education level. The indexes of ∆CFI, ∆TLI, and ∆RMSEA indicated the equivalence of the three-factor structure of the simplified version between groups. However, *χ*^2^ might be significant in model fit across groups due to large sample size (61).

**Table 5:** Invariance of the simplified version of the Chinese resident health literacy scale across groups of gender, age, cognitive status, and educational levels in Sample B.

Groups	Invariance levels	*χ*^2^	df	*p-*value	Δ*χ^2^*	Δdf	*p* (Δ*χ*^2^)	CFI	ΔCFI	TLI	ΔTLI	GFI	ΔGFI	RMSEA	ΔRMSEA
Gender															
	Male	1759.06	319	<0.001				0.813		0.794		0.902		0.056	
	Female	1719.63	319	<0.001				0.831		0.814		0.903		0.055	
	Configural	3478.70	638	<0.001				0.822		0.805		0.902		0.039	
	Metric	3505.46	662	<0.001	26.76	24	0.316	0.822	0.000	0.811	0.006	0.902	0.000	0.038	0.001
	Strong	3508.39	668	<0.001	2.93	6	0.818	0.822	0.000	0.813	0.002	0.902	0.000	0.038	0.000
	Strict	3539.81	697	<0.001	31.42	29	0.346	0.822	0.000	0.821	0.008	0.901	0.001	0.037	0.001
Age															
	60–69	1709.95	319	<0.001				0.824		0.806		0.902		0.055	
	≥70 or over	1719.84	319	<0.001				0.827		0.809		0.904		0.055	
	Configural	3429.78	638	<0.001				0.825		0.808		0.903		0.039	
	Metric	3452.84	662	<0.001	23.06	24	0.516	0.825	0.000	0.815	0.007	0.903	0.000	0.038	0.001
	Strong	3464.03	668	<0.001	11.19	6	0.083	0.825	0.000	0.816	0.001	0.903	0.000	0.038	0.000
	Strict	3508.25	697	<0.001	44.22	29	0.035	0.824	0.001	0.823	0.007	0.902	0.001	0.037	0.001
Cognitive status															
	Normal	2292.98	319	<0.001				0.810		0.791		0.905		0.056	
	Impairment	1285.07	319	<0.001				0.816		0.798		0.892		0.056	
	Configural	3578.11	638	<0.001				0.812		0.793		0.901		0.040	
	Metric	3631.58	662	<0.001	53.47	24	0.001	0.810	0.002	0.799	0.006	0.900	0.001	0.039	0.001
	Strong	3647.84	668	<0.001	16.26	6	0.012	0.810	0.000	0.800	0.001	0.899	0.001	0.039	0.000
	Strict	3884.22	697	<0.001	236.38	29	0.000	0.796	0.004	0.795	0.005	0.893	0.006	0.040	0.001
Education level															
	Education 1	1262.39	319	<0.001				0.820		0.802		0.882		0.060	
	Education 2	1379.87	319	<0.001				0.794		0.773		0.897		0.056	
	Education 3	1313.93	319	<0.001				0.809		0.789		0.900		0.056	
	Configural	4394.94	1,016	<0.001				0.783		0.775		0.883		0.034	
	Metric	4425.02	1,040	<0.001	30.08	24	0.182	0.783	0.000	0.780	0.005	0.882	0.001	0.033	0.001
	Strong	4430.57	1,046	<0.001	5.55	6	0.475	0.783	0.000	0.781	0.001	0.882	0.000	0.033	0.000
	Strict	4471.92	1,075	<0.001	41.35	29	0.064	0.782	0.001	0.787	0.006	0.881	0.001	0.033	0.000

*χ*^2^: chi-square; df: degrees of freedom; CFI, Comparative fit index; TLI: Tucker–Lewis index; GFI, Goodness of Fit Index; RMSEA, root mean square error of approximation.

Education 1: Illiterate or Primary school; Education 2: Junior school; Education 3: High school, College, or Graduate school.

## Discussion

In this study, we first simplified the CRHLS (2018 edition) based on IRT and DIF methods. Finally, the simplified version of the remaining 27 items for older adults was adapted with a good reliability and even slightly improved in construct validation. The original scale, widely used in the periodic national survey to monitor the level and dynamic changes of health literacy in Chinese adults aged 15–69 years old, was developed on the manual of “Basic Knowledge and Skills of People’s Health Literacy” published by the National Health and Family Planning Commission (18), which was still an important material of health education and health communication for the public in China now. Therefore, this simplified version adopted from CRHLS focused on older adults might be more suitable for extended tracking of the dynamic changes in the whole life cycle in the future, evaluating the intervention effect of health education in chronic diseases, and investigating the relationship to health outcomes or health behaviors under the Chinese context. Moreover, it was more convenient for collection and administration of data than the original CRHLS.

Recently, IRT was more and more applied to assess and revise the scales in public health or clinical studies (43). Different from the previous simplified studies just focused on the difficulty and discrimination when using IRT model (31, 62–64), our findings also integrated the equivalence and information of items into the criteria of remaining items in order to obtain a relative accuracy of measurement of health literacy for older adults. The previous study by Shen et al. reported the overall reliability of original version was 0.95 among the population aged 15–69 years old in China (16). In contrast, the reliability of original version in our study was found to be 0.90 for the older adults, but still a good reliability. It also showed that original CRHLS, although with some shortcomings, could be used to measure the level of health literacy for the old population. Compared to the original version, simplified version still had good overall reliability ( α=0.87 ) and acceptable reliability in each dimension, which was slightly lower due to the great reduction of items. Moreover, according to the Goodness-of-fits in three factors models of CFA in our study, the simplified version seemed to be better construct validation and construct invariance across groups. All of these supported that the simplified version scale might be suitable for the priority recommendation as an instrument of health literacy for older adults in Chinese social context.

We simplified the Chinese instrument for measuring health literacy by removing some redundant or DIF items to finally form the simplified version with 27 items focused on older adults in China. However, there were some limitations. Firstly, IRT model needed to meet basic assumptions of unidimensionality, monotonicity and local independence. In this study, the data was verified to satisfy these basic assumptions only except for the eigenvalue examination in the dimension of health-related skills (the ratio of the first to second eigenvalue was 2.52, which slightly less than 3 of threshold value). Secondly, because the original version was initially developed by an expert panel from the Ministry of Health and now was widely used for community-based adults aged 15–69 years old in China, we did not further assess the content validity in this study. Moreover, the items kept in short version were selected based on IRT and DIF. The additional items recommended by researchers might be subjective, so did not be considered in this study. Thirdly, we used the data of the representative sample of older adults from a cross-sectional study conducted in Beijing. There might be differences in characteristics among older adults living in different regions, which might have an impact on the reliability and validity of the scale when it was extended used in general Chinese older adults. So, this simplified version needs to the further assess in the general populations.

## Conclusion

In this study, we form a simplified version instrument for measuring health literacy focused on older adults in China. It might be more suitable for the priority recommendation in extended tracking of the dynamic changes in the whole life cycle and assessing the level of health literacy among older adults in public health settings.

## Data availability statement

The data analyzed in this study is subject to the following licenses/restrictions: The raw data supporting the conclusions of this article will be made available by the authors, without undue reservation. Requests to access these datasets should be directed to YHW, wyhong826@pumc.edu.cn.

## Ethics statement

The studies involving human participants were reviewed and approved by the institutional review board of the institute of Basic Medical Sciences, Chinese Academy of Medical Sciences (Project No. 064–2020). The patients/participants provided their written informed consent to participate in this study.

## Author contributions

YHW and LW participated in the design of the study and organized the training of investigators. QJ, HW, YLW, and KZ participated in data collection and quality control. BY and LL participated in the literature research and collation. YLW and YHW drafted the manuscript, performed the statistical analysis, and revision of the manuscript. All authors contributed to the article and approved the submitted version.

## Funding

The survey was funded by National Key R&D Program of China (2022YFC3601800), Beijing Municipal Health Commission and Beijing Health Economics Association. The funders had no role in the design of the study, analysis, and interpretation of data, or writing the manuscript.

## Conflict of interest

The authors declare that the research was conducted in the absence of any commercial or financial relationships that could be construed as a potential conflict of interest.

## Publisher’s note

All claims expressed in this article are solely those of the authors and do not necessarily represent those of their affiliated organizations, or those of the publisher, the editors and the reviewers. Any product that may be evaluated in this article, or claim that may be made by its manufacturer, is not guaranteed or endorsed by the publisher.
