# Supplementary material for: Revised Chinese resident health literacy scale for the older adults in China: simplified version and initial validity testing
Source: Front Public Health. 2023 May 17;11:1147862. doi: 10.3389/fpubh.2023.1147862 (PMC10231683; doi:10.3389/fpubh.2023.1147862)
Supplement: Supplementary file 1 [file Data_Sheet_1.pdf]

Supplementary Material

Revised Chinese Resident Health Literacy Scale for Older Adults in China: Simplified Version and Initial Validity Testing

Yilin Wang[1], Qiaoling Jia[1], Haiyan Wang[1], Kaiwen Zou[1], Lu Li[1], Bing Yu[1], Li Wang[1] and Yanhong Wang[1*]

[1] Department of Epidemiology and Biostatistics, Institute of Basic Medical Sciences, Academy of Medical Sciences & School of Basic Medicine Peking Union Medical College, 5 Dong Dan San Tiao, Beijing, China

*** Correspondence:**
Yanhong Wang
wyhong826@pumc.edu.cn

Supplementary Table 1. Evaluation of items of the short version of the Chinese Resident Health Literacy Scale in Sample B

Item abbreviation		Correct (%)	Item correlation to dimension score	Factor Loading
Dimension1: Knowledge and attitudes				
A01	*Prevention of the flu*	79.00	0.44	0.32
A03	*Infusion*	79.34	0.44	0.28
B01	*The definition of health*	72.51	0.55	0.46
B07	*Management of gas poisoning*	77.87	0.43	0.34
B09	*Toxic and hazardous work*	70.97	0.50	0.39
B17	*Meaning of warning diagram*	69.94	0.56	0.43
C02	*Medical visits*	70.25	0.51	0.46

C07	*Treatment of sick and dead livestock*	65.96	0.55	0.54
C15	*Pesticide storage*	58.06	0.55	0.56
D03	*Control weight*	61.63	0.55	0.47
D04	*Obesity-related disease*	66.51	0.52	0.42
Dimension2: Behavior and lifestyles				
B05	*Dangers of smoking*	50.55	0.53	0.38
B12	*National basic public health service*	38.33	0.52	0.42
B19	*Medical visits*	70.38	0.58	0.48
B21	*Opening windows for ventilation during flu season*	69.32	0.53	0.39
C01	*Promoting mental health*	49.42	0.66	0.60
C04	*Fever and rash in children*	56.76	0.65	0.62
C09	*Benefits of eating soy products*	32.29	0.50	0.40
C10	*Health benefits of exercise*	44.51	0.62	0.55
C13	*Medical visits*	67.30	0.50	0.48
Dimension3: Health-related skills				
B16	*Treatment of virulent infectious diseases*	83.67	0.50	0.41
C08	*Cardiac arrest*	57.28	0.62	0.35
C11	*Hypoglycemic products*	71.62	0.54	0.37
C14	*Benefits of breastfeeding for babies*	43.24	0.61	0.50
C16	*Lightning weather outdoors*	82.36	0.46	0.41
D01	*Calculation of BMI*	33.36	0.60	0.52
D02	*Classification of BMI*	42.76	0.63	0.42

Note. The correlation coefficient between the three dimensions of the short version were: 0.66 between Dimension1 and Dimension2; 0.71 between Dimension1 and Dimension3; 0.65 between Dimension2 and Dimension3.
