# Supplementary material for: Revised Chinese resident health literacy scale for the older adults in China: simplified version and initial validity testing
Source: Front Public Health. 2023 May 17;11:1147862. doi: 10.3389/fpubh.2023.1147862 (PMC10231683; doi:10.3389/fpubh.2023.1147862)
Supplement: Supplementary file 2 [file Table_1.DOCX]

Supplementary Material

**Revised Chinese Resident Health Literacy Scale for Older Adults in China:**

**Simplified Version and Initial Validity Testing**

Yilin Wang^1^, Qiaoling Jia^1^, Haiyan Wang^1^, Kaiwen Zou^1^, Lu Li^1^, Bing Yu^1^, Li Wang^1^ and Yanhong Wang^1*^

^1^ Department of Epidemiology and Biostatistics, Institute of Basic Medical Sciences, Academy of Medical Sciences & School of Basic Medicine Peking Union Medical College, 5 Dong Dan San Tiao, Beijing, China

*** Correspondence:**Yanhong Wang
[wyhong826@pumc.edu.cn](mailto:wyhong826@pumc.edu.cn)

**Supplementary Table 1.** Evaluation of items of the short version of the Chinese Resident Health Literacy Scale in Sample B

| Item abbreviation | | Correct (%) | Item correlation to  dimension score | Factor Loading |
| --- | --- | --- | --- | --- |
| Dimension1: Knowledge and attitudes | | | |  |
| A01 | *Prevention of the flu* | 79.00 | 0.44 | 0.32 |
| A03 | *Infusion* | 79.34 | 0.44 | 0.28 |
| B01 | *The definition of health* | 72.51 | 0.55 | 0.46 |
| B07 | *Management of gas poisoning* | 77.87 | 0.43 | 0.34 |
| B09 | *Toxic and hazardous work* | 70.97 | 0.50 | 0.39 |
| B17 | *Meaning of warning diagram* | 69.94 | 0.56 | 0.43 |
| C02 | *Medical visits* | 70.25 | 0.51 | 0.46 |
| C07 | *Treatment of sick and dead livestock* | 65.96 | 0.55 | 0.54 |
| C15 | *Pesticide storage* | 58.06 | 0.55 | 0.56 |
| D03 | *Control weight* | 61.63 | 0.55 | 0.47 |
| D04 | *Obesity-related disease* | 66.51 | 0.52 | 0.42 |
| Dimension2: Behavior and lifestyles | | | |  |
| B05 | *Dangers of smoking* | 50.55 | 0.53 | 0.38 |
| B12 | *National basic public health service* | 38.33 | 0.52 | 0.42 |
| B19 | *Medical visits* | 70.38 | 0.58 | 0.48 |
| B21 | *Opening windows for ventilation during flu season* | 69.32 | 0.53 | 0.39 |
| C01 | *Promoting mental health* | 49.42 | 0.66 | 0.60 |
| C04 | *Fever and rash in children* | 56.76 | 0.65 | 0.62 |
| C09 | *Benefits of eating soy products* | 32.29 | 0.50 | 0.40 |
| C10 | *Health benefits of exercise* | 44.51 | 0.62 | 0.55 |
| C13 | *Medical visits* | 67.30 | 0.50 | 0.48 |
| Dimension3: Health-related skills | | | |  |
| B16 | *Treatment of virulent infectious diseases* | 83.67 | 0.50 | 0.41 |
| C08 | *Cardiac arrest* | 57.28 | 0.62 | 0.35 |
| C11 | *Hypoglycemic products* | 71.62 | 0.54 | 0.37 |
| C14 | *Benefits of breastfeeding for babies* | 43.24 | 0.61 | 0.50 |
| C16 | *Lightning weather outdoors* | 82.36 | 0.46 | 0.41 |
| D01 | *Calculation of BMI* | 33.36 | 0.60 | 0.52 |
| D02 | *Classification of BMI* | 42.76 | 0.63 | 0.42 |

*Note. The correlation coefficient between the three dimensions of the short version were: 0.66 between Dimension1 and Dimension2;*

*0.71 between Dimension1 and Dimension3; 0.65 between Dimension2 and Dimension3.*
